# Supplementary material for: Adolescent Mental Health during the COVID-19 Pandemic: The Interplay of Age, Gender, and Mental Health Outcomes in Two Consecutive Cross-Sectional Surveys in Northern Italy
Source: Behav Sci (Basel). 2023 Aug 1;13(8):643. doi: 10.3390/bs13080643 (PMC10451173; doi:10.3390/bs13080643)
Supplement: Supplementary file 1 [file behavsci-13-00643-s001.zip › behavsci-2529043-supplementary.pdf]

**Figure S1.** Distribution of combinations of adolescent reports of symptom of anxiety (SCARED), depressive symptoms (PHQ-2), health-related quality of life (HRQoL) and at least 3 different psychosomatic symptoms a week (HBSC) per COP-S 2021 (Survey 1) of COP-S 2022 (Survey 2) and gender.

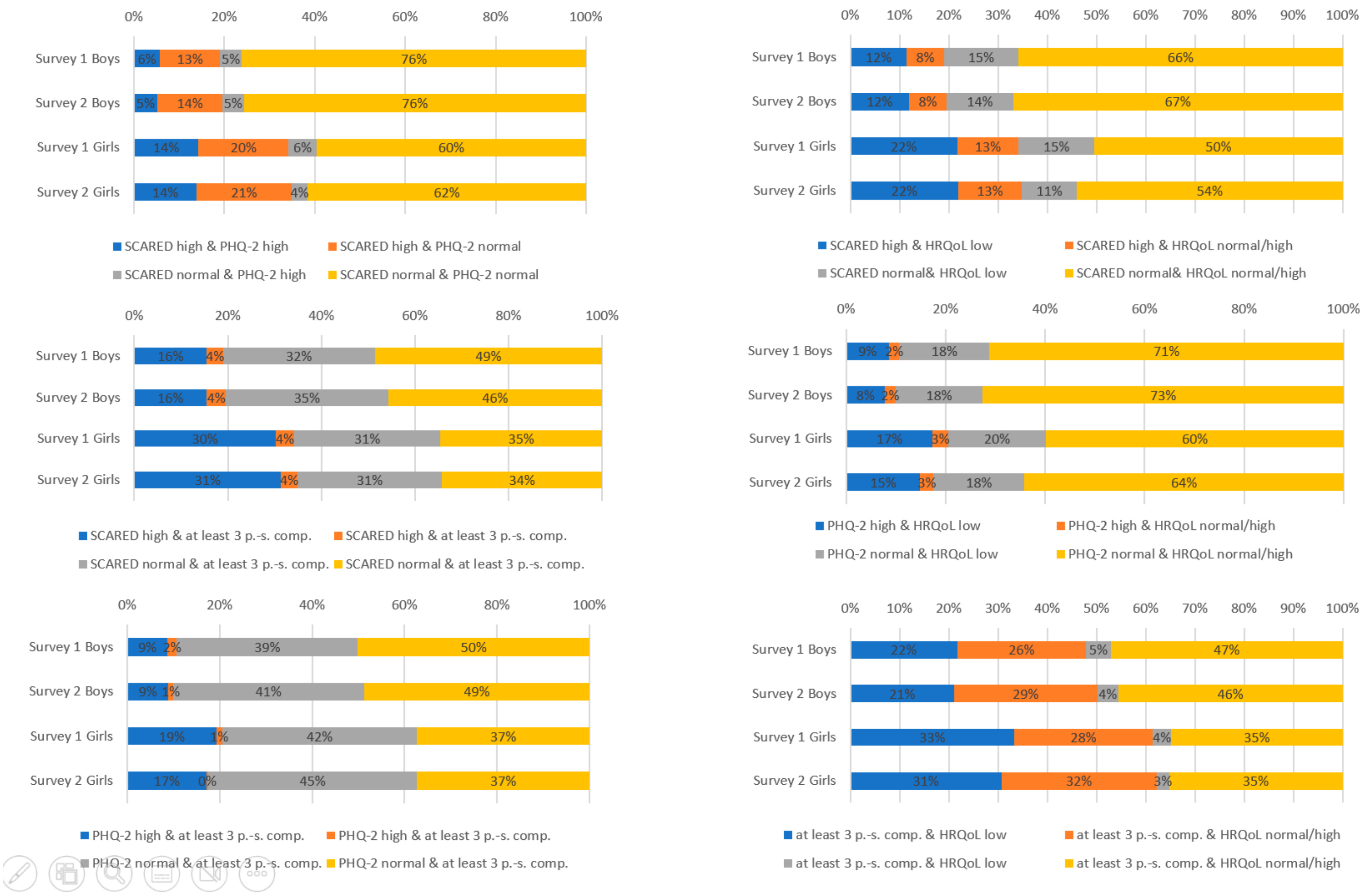

**Table S1.** Prediction of anxiety, depressive symptoms, and low HRQoL in adolescent boys and girls in 2021 and 2022, identified by forward regression analysis using psychosomatic complaints as significant predictor

| Outcome | COPS-S | Gender | Predictor | OR | 95% CI | *p*–value |
|---|---|---|---|---|---|---|
| Anxiety | 2021 | Boys | Higher number of psychosomatic complaints | 1.582 | [1.444;1.731] | < 0.001 |
| | | Girls | Higher number of psychosomatic complaints | 1.565 | [1.452;1.692] | < 0.001 |
| | | | Higher sum score of pandemic related burden | 1.130 | [1.041;1.243] | 0.004 |
| | 2022 | Boys | Higher number of psychosomatic complaints | 1.465 | [1.341;1.595] | < 0.001 |
| | | | Lower family climate due to the pandemic | 1.695 | [1.085;2.647] | 0.02 |
| | | | Higher parental workload due to the pandemic | 1.876 | [1.285;2.741] | 0.001 |
| | | Girls | Higher number of psychosomatic complaints | 1.633 | [1.511;1.768] | < 0.001 |
| | | | Low family climate | 1.823 | [1.275;2.806] | < 0.001 |
| | | | Higher sum score of pandemic related burden | 1.177 | [1.063;1.289] | 0.001 |
| Depressive symptoms | 2021 | Boys | Higher number of psychosomatic complaints | 1.522 | [1.347;1.706] | < 0.001 |
| | | | Higher age | 1.165 | [1.014;1.347] | < 0.001 |
| | | | Low family climate | 2.810 | [1.657;4.666] | < 0.001 |
| | | Girls | Higher number of psychosomatic complaints | 1.731 | [1.552;1.933] | < 0.001 |
| | | | Higher sum score of pandemic related burden | 1.232 | [1.097;1.381] | < 0.001 |
| | | | Low family climate | 2.475 | [1.580;3.760] | < 0.001 |
| | | | Higher age | 1.097 | [1.003;1.207] | 0.044 |
| | 2022 | Boys | Higher number of psychosomatic complaints | 1.597 | [1.421;1.786] | < 0.001 |
| | | | Higher age | 1.178 | [1.062;1.315] | 0.002 |
| | | Girls | More psychosomatic complaints | 2.099 | [1.851;2.380] | < 0.001 |
| | | | Low family climate | 2.287 | [1.495;3.649] | < 0.001 |
| Low HRQoL | 2021 | Boys | Higher number of psychosomatic complaints | 1.569 | [1.425;1.722] | < 0.001 |
| | | | Low family climate | 3.113 | [2.038;4.728] | < 0.001 |
| | | | Higher sum score of pandemic related burdens | 1.360 | [1.217;1.516] | < 0.001 |
| | | | Higher age | 1.198 | [1.098;1.302] | < 0.001 |
| | | Girls | Higher number of psychosomatic complaints | 1.741 | [1.578;1.912] | < 0.001 |

| | | | | | |
|---|---|---|---|---|---|
| | | Higher sum score of pandemic related burden | 1.547 | [1.391;1.757] | < 0.001 |
| | | Low family climate | 4.209 | [2.636;6.151] | < 0.001 |
| | | Lower sociodemographic conditions | 3.369 | [1.349;8.409] | 0.009 |
| | | Higher age | 1.120 | [1.031;1.220] | < 0.001 |
| 2022 | Boys | Higher number of psychosomatic complaints | 1.600 | [1.466;1.761] | < 0.001 |
| | | Lower family climate due to the pandemic | 2.788 | [1.774;4.427] | < 0.001 |
| | | Higher sum score of pandemic related burden | 1.303 | [1.162;1.463] | < 0.001 |
| | | Higher age | 1.150 | [1.063;1.254] | 0.001 |
| | | Higher parental workload due to the pandemic | 1.680 | [1.120;2.439] | 0.011 |
| | Girls | Higher number of psychosomatic complaints | 1.782 | [1.622;1.947] | < 0.001 |
| | | Low family climate | 2.592 | [1.666;1.947] | < 0.001 |
| | | Higher age | 1.170 | [1.079;1.278] | < 0.001 |
| | | Higher sum score of pandemic related burden | 1.477 | [1.322;1.657] | < 0.001 |

HRQoL, Health-related quality of life; CI, confidence interval; OR, odds ratio

**Table S2.** Prediction of low HRQoL, depressive symptoms, and psychosomatic complaints in adolescent boys and girls in 2021 and 2022, identified by forward regression analysis using anxiety sum score as significant predictor

| Outcome | COP-S | Gender | Predictor | OR | 95% CI | p–value |
|---|---|---|---|---|---|---|
| Low HRQoL | 2021 | Boys | Anxiety sum score | 1.309 | (1.239;1.382) | < 0.001 |
| | | | Low family climate te | 3.832 | (2.492;5.895) | < 0.001 |
| | | | Pandemic burden | 1.352 | (1.227;1.362) | < 0.001 |
| | | | Age | 1.237 | (1.134;1.349) | < 0.001 |
| | | Girls | Anxiety sum score | 1.212 | (1.163;1.262) | < 0.001 |
| | | | Pandemic burden | 1.451 | (1.303;1.616) | < 0.001 |
| | | | Low family climate | 5.111 | (3.429;7.617) | < 0.001 |
| | | | Socioeconomic Conditions | 3.888 | (1.623;9.313) | 0.002 |
| | | | Age | 1.183 | (1.093;1.280) | < 0.001 |
| | 2022 | Boys | Anxiety sum score | 1.309 | (1.242;1.382) | < 0.001 |
| | | | Low family climate | 3.140 | (2.002;5.024) | < 0.001 |
| | | | Pandemic burden | 1.312 | (1.164;1.471) | < 0.001 |
| | | | Age | 1.170 | (1.078;1.270) | 0.001 |
| | | | Parental Workload | 1.635 | (1.091;2.365) | 0.016 |
| | | Girls | Anxiety sum score | 1.244 | (1.196;1.295) | < 0.001 |
| | | | Low family climate | 2.990 | (1.977;4.522) | < 0.001 |
| | | | Age | 1.240 | (1.143;1.341) | < 0.001 |
| | | | Pandemic burden | 1.385 | (1.253;1.548) | < 0.001 |
| Depressive symptoms | 2021 | Boys | Anxiety sum score | 1.272 | (1.196;1.352) | < 0.001 |
| | | | Low family climate | 2.947 | (1.738;4.997) | < 0.001 |
| | | | Age | 1.174 | (1.054;1.308) | 0.004 |
| | | Girls | Anxiety sum score | 1.241 | (1.186;1.300) | < 0.001 |
| | | | Pandemic burden | 1.169 | (1.043;1.310) | 0.007 |
| | | | Low family climate | 3.426 | (2.247;5.225) | < 0.001 |
| | | | Age | 1.171 | (1.070;1.281) | < 0.001 |
| | 2022 | Boys | Anxiety sum score | 1.231 | (1.164;1.302) | < 0.001 |
| | | | Age | 1.172 | (1.057;1.300) | 0.003 |
| | | Girls | Anxiety sum score | 1.307 | (1.246;1.370) | < 0.001 |
| | | | Low family climate | 2.582 | (1.673;4.522) | < 0.001 |
| | | | Age | 1.143 | (1.038;1.258) | < 0.001 |

| | | | | | | |
|---|---|---|---|---|---|---|
| At least three psychosomatic complaints | 2021 | Boys | Anxiety sum score | 1.343 | (1.275;1.415) | < 0.001 |
| | | | Low family climate | 3.474 | (2.313;5.218) | < 0.001 |
| | | | Age | 1.098 | (1.023;1.179) | < 0.01 |
| | | Girls | Anxiety sum score | 1.279 | (1.240;1.319) | < 0.001 |
| | | | Low family climate | 2.134 | (1.418;3.211) | < 0.001 |
| | | | Age | 1.117 | (1.039;1.201) | 0.003 |
| | 2022 | Boys | Anxiety sum score | 1.297 | (1.236;1.360) | < 0.001 |
| | | | Low family climate | 2.775 | (1.746;4.413) | < 0.001 |
| | | Girls | Anxiety sum score | 1.308 | (1.251;1.368) | < 0.001 |
| | | | Low family climate | 2.486 | (1.541;4.011) | < 0.001 |
| | | | Age | 1.129 | (1.051;1.212) | 0.001 |

HRQoL, Health-related quality of life; CI, confidence interval; OR, odds ratio

**Table S3.** Prediction of low HRQoL, symptoms of anxiety, and psychosomatic complaints in adolescent boys and girls in 2021 and 2022, identified by forward regression analysis using depressive symptoms score as significant predictor

| Symptom | Year | Gender | Predictor | OR | 95% CI | p-value |
|---|---|---|---|---|---|---|
| Low HRQoL | 2021 | Boys | Depressive symptoms sum score | 2.623 | (2.160;3.187) | < 0.001 |
| | | | Low family climate | 2.837 | (1.829;4.402) | < 0.001 |
| | | | Pandemic burden | 1.307 | (1.169;1.461) | < 0.001 |
| | | | Parental workload | 1.541 | (1.025;2.314) | < 0.001 |
| | | | Age | 1.144 | (1.048;1.248) | < 0.01 |
| | | Girls | Depressive symptoms sum score | 2.730 | (2.282;3.266) | < 0.001 |
| | | | Low family climate | 3.241 | (2.129;4.934) | < 0.001 |
| | | | Pandemic burden | 1.431 | (1.276;1.605) | < 0.001 |
| | | | Age | 1.118 | (1.025;1.219) | 0.012 |
| | 2022 | Boys | Depressive symptoms sum score | 3.190 | (2.645;3.847) | < 0.001 |
| | | | Low family climate | 2.336 | (1.491;3.660) | < 0.001 |
| | | | Pandemic burden | 1.241 | (1.101;1.399) | < 0.001 |
| | | | Parental workload | 1.980 | (1.315;2.981) | 0.001 |
| | | Girls | Depressive symptoms sum score | 3.190 | (2.645;3.847) | < 0.001 |
| | | | Low family climate | 2.336 | (1.491;3.660) | < 0.001 |
| | | | Pandemic burden | 1.451 | (1.290;1.632) | < 0.001 |
| | | | Age | 1.134 | (1.040;1.237) | 0.005 |
| Symptoms of Anxiety | 2021 | Boys | Depressive symptoms sum score | 2.090 | (1.798;2.428) | < 0.001 |
| | | Girls | Depressive symptoms sum score | 2.084 | (1.838;2.363) | < 0.001 |
| | 2022 | Boys | Depressive symptoms sum score | 1.995 | (1.707;2.331) | < 0.001 |
| | | | Low family climate | 1.735 | (1.098;2.741) | < 0.001 |
| | | | Parental workload | 2.022 | (1.375;2.973) | < 0.001 |
| | | | Age | 0.900 | (0.827;0.979) | 0.014 |

| | | | | | | |
|---|---|---|---|---|---|---|
| | | Girls | Depressive symptoms sum score | 2.246 | (1.952;2.585) | < 0.001 |
| | | | Low family climate | 1.845 | (1.238;2.750) | 0.003 |
| | | | Pandemic burden | 1.143 | (1.037;1.260) | 0.003 |
| At least three psychosomatic symptoms | 2021 | Boys | Depressive symptoms sum score | 2.452 | (2.051;2.931) | < 0.001 |
| | | | Low family climate | 2.788 | (1.867;4.165) | < 0.001 |
| | | Girls | Depressive symptoms sum score | 3.408 | (2.812;4.129) | < 0.001 |
| | 2022 | Boys | Depressive symptoms sum score | 2.906 | (2.410;3.504) | < 0.001 |
| | | | Low family climate | 2.569 | (1.596;4.135) | < 0.001 |
| | | Girls | Depressive symptoms sum score | 4.289 | (3.443;5.342) | < 0.001 |
| | | | Low family climate | 1.943 | (1.173;3.221) | 0.01 |

HRQoL, Health-related quality of life; CI, confidence interval; OR, odds ratio

**Table S4.** Predictors of elevated symptoms of anxiety, elevated depressive symptoms and low HRQoL self -reported by adolescents in the second and the third year of the pandemic, self-reported anxiety included as independent predictor

| Outcome | Gender | COP-S | Intercept [#] | Age [#] | Sociodemographic condition [#,†] | Parent's burden [#,‡] | Child's burden [#,§] | Low family climate [#] | Anxiety sum score [#] | Model fit (Nagelkerke's $R^2$) |
|---|---|---|---|---|---|---|---|---|---|---|
| Low HRQoL | Boys | 2021 | 0.000*** | 1.237*** | | | 1.352*** | 3.832** | 1.309** | 0.439 |
| | | 2022 | 0.001*** | 1.170*** | | 1.635 | 1.312*** | 3.140*** | 1.309*** | 0.392 |
| | Girls | 2021 | 0.000*** | 1.183*** | 3.888* | | 1.451*** | 5.111*** | 1.212*** | 0.469 |
| | | 2022 | 0.000*** | 1.240*** | | | 1.385*** | 2.990*** | 1.244*** | 0.455 |
| Depressive symptoms | Boys | 2021 | 0.004*** | 1.181** | | | | 2.995** | 1.274*** | 0.289 |
| | | 2022 | 0.007*** | 1.170*** | | | | | 1.233*** | 0.159 |
| | Girls | 2021 | 0.001*** | 1.167*** | | | 1.176** | 3.500** | 1.237*** | 0.376 |
| | | 2022 | 0.005** | 1.140** | | | | 2.561*** | 1.308*** | 0.396 |
| At least three psycho-somatic complaints | Boys | 2021 | 0.081*** | 1.098** | | | | 3.474*** | 1.343** | 0.361 |
| | | 2022 | 0.305*** | | | | | 2.775*** | 1.297*** | 0.288 |
| | Girls | 2021 | 0.086*** | 1.117** | | | | 2.134*** | 1.297*** | 0.358 |
| | | 2022 | 0.103*** | 1.129*** | | | | 2.486** | 1.308*** | 0.366 |

[#] The table indicates logistic regression odds ratios for all significant independent variables, controlling for the other predictors. * $p < 0.05$, ** $p < 0.01$, *** $p < 0.005$.

[†] Single parenthood OR low parental education OR parental mental health problems (dichotomous).

[‡] Extended parents' burden due to pandemic (dichotomous).

[§] Child's pandemic-related burden due school, less contact with friends, extended use of digital media.

**Table S5.** Predictors of low HRQoL, elevated depressive symptoms, and weekly psychosomatic complaints elevated symptoms of anxiety, and self -reported by adolescents in the second and the third year of the pandemic, self-reported depressive symtoms included as independent predictor

| Outcome | Gender | COP-S | Intercept [#] | Age [#] | Sociodemographic condition [#,†] | Parent's burden [#,‡] | Child's burden [#,§] | Low family climate [#] | Depressive symptoms sum score [#] | Model fit (Nagelkerke's $R^2$) |
|---|---|---|---|---|---|---|---|---|---|---|
| Low HRQoL | Boys | 2021 | 0.001*** | 1.145** | | 1.543** | 1.308*** | 2.838*** | 2.630*** | 0.465 |
| | | 2022 | 0.005*** | | | 1.980** | 1.252*** | 2.855*** | 3.190*** | 0.454 |
| | Girls | 2021 | 0.001*** | 1.117* | | | 1.412*** | 3.450*** | 2.721*** | 0.564 |
| | | 2022 | 0.000*** | 1.139** | | | 1.437*** | 2.386*** | 3.170*** | 0.556 |
| Elevated symptoms of anxiety | Boys | 2021 | 0.089*** | | | | | | 2.084*** | 0.218 |
| | | 2022 | 0.163*** | 0.896* | | 2.017*** | | 1.735* | 1.996*** | 0.234 |
| | Girls | 2021 | 0.172*** | | | | | | 2.069*** | 0.277 |
| | | 2022 | 0.032*** | | | | 1.145** | 1.780** | 2.247*** | 0.363 |
| At least three psycho-somatic complaints | Boys | 2021 | 0.357*** | | | | | 2.757*** | 2.704*** | 0.313 |
| | | 2022 | 0.173*** | | | | | 2.569*** | 2.898*** | 0.338 |
| | Girls | 2021 | 0.413*** | | | | | | 3.446*** | 0.401 |
| | | 2022 | 0.382*** | | | | | 1.883*** | 4.201*** | 0.44 |

[#] The table indicates logistic regression odds ratios for all significant independent variables, controlling for the other predictors. * $p < 0.05$, ** $p < 0.01$, *** $p < 0.005$.

[†] Single parenthood OR low parental education OR parental mental health problems (dichotomous).

[‡] Extended parents' burden due to pandemic (dichotomous).

[§] Child's pandemic-related burden due school, less contact with friends, extended use of digital media.
